# Germany vs. Austria: country-specific differences in becoming parents during the COVID-19 pandemic

**DOI:** 10.1093/eurpub/ckac131.447

**Published:** 2022-10-25

**Authors:** J Preiß, C Florea, M Angerer, M Schabus

**Affiliations:** 1 Laboratory for Sleep & Consciousness Research, University of Salzburg, Salzburg, Austria; 2 Centre for Cognitive Neuroscience Salzburg, University of Salzburg, Salzburg, Austria

## Abstract

**Background:**

Becoming parents can be a big challenge, but how is this experience affected by the COVID-19 pandemic and its measures?

**Methods:**

Between 18.05.2021 and 01.07.2021, we conducted an online-survey to gain insight into becoming parents during the COVID-19 pandemic in Germany and Austria. The sample mentioned in this report consists of biological mothers living in Austria (n = 952) and Germany (n = 1012) who gave birth between the 16th of March 2020 (the beginning of the first lockdown in Austria) and the time of completion of the study. The mothers’ current stress levels were assessed with the Perceived Stress Scale (PSS), and postnatal depression symptoms were quantified with the Edinburgh Postnatal Depression Scale (EPDS). Furthermore, we included questions to measure perceived social support and pandemic-related stress.

**Results:**

Current stress levels (U = 555677.50, z = 5.90, p < .001) and postnatal depression symptoms (U = 546354.00, z = 5.15, p < .001) were significantly higher in the German sample as compared to the Austrian sample. Yet, Austrian mothers reported higher social support as compared to the German mothers (U = 387834.00, z = -7.48, p < .001). Furthermore, we found higher levels of perceived social support to be associated with lower current stress levels (rs = -.40, p < .001). On average, perceived stress was moderately high in both samples (MGermany = 18.58, SDGermany = 6.60; MAustria = 16.76, SDAustria = 6.57).

**Conclusions:**

Stress levels and depression symptoms seem to be high and prevalent due to the pandemic and it is highly indicated to take action such as supporting families in need and emphasising social support in order to reduce mental health problems of parents as well as their children in the aftermath of the pandemic.

**Key messages:**

• Findings underpin the protective role of social support against psychological distress in new parents and show the high strain at current.

• Action needs to be taken in order to support parents and children at risk.

